# The effect of hydro-alcoholic of *Phoenix Dactylifera* extract on sleep and EEG in rat 

**Published:** 2017

**Authors:** Sahar Rahimi, Hojjatollah Alaei, Parham Reisi, Behzad Zolfaghari, Zahra Siahmard, Aliasghar Pourshanazari

**Affiliations:** 1 *Department of Physiology, School of Medicine, Isfahan University of Medical Science, Isfahan, Iran*; 2 *Department of Pharmacognosy, School of Pharmacy, Isfahan University of Medical Science, Isfahan, Iran*

**Keywords:** Insomnia, Sleep, EEG, Tarooneh

## Abstract

**Objective:**

Hard envelope of date palm pollen is used as a sedative and calmative compounds in Iranian traditional medicine. We tried to study the effects of *Phoenix dactylifera* (Tarooneh) extract on sleep time and Brian waves.

**Materials and Methods:**

Rats were divided into control and test groups in sleep experiment. Control groups included intact group (without any injection), negative control group (saline) and positive control group (midazolam 0.1 mg/kg). Test groups received three doses of Tarooneh extract (62.5, 125 and 250 mg/kg). Rat were placed in sleep physiograph system and recording started 20 min after 2-hr calming down. Four parameters including sleep time, awakening, most activity period and main sleep time interval were measured. In EEG experiment electrodes were placed under the cranium for EEG recording and waves were compared with their baselines.

**Results:**

All doses of the extract increased sleeping time (p< 0.05) but just the dose of 250 mg/kg (p<0.05) and midazolam (p< 0.001) decreased the awakening time. EEG results showed that the dose of 125 mg/kg increased the low frequency waves (p< 0.05) and the dose of 250 mg/kg decreased high frequency waves of alpha and beta (p< 0.05).

**Conclusion:**

Due to these effect on sleeping time and EEG, Tarooneh extract consumption can be useful as a sedative agent in Iranian traditional medicine. According to this study, the doses of 125 and 250 mg/kg of the extract would be the appropriate doses to be further studied.

## Introduction

Sleep is a repetitive cycle in life that helps saving body energy and improving sleep problems, which occur when the duration or quality of sleeping is disturbed, cause health problems (Abram, 2015 ▶) and the concomitant mental and physical disorders (Budhiraja et al., 2014 ▶). People suffering from sleep problems are also at high risk for cognitive disorders (Yaffe et al., 2011 ▶). During sleep, brain wave activity has less frequency which is concomitant with getting in deeper stages of sleep although in eliminated sensory input, there is extended brain structure and activity like neocortex changes that exist in NREM (Non Rapid Eye Movement) sleep (David et al., 2013 ▶). Sleep spindles and delta brain waves are common NREM signs and 0.5-1 Hz brain wave activities show an important specific sign of NREM sleep (Steriade, 2006 ▶). Many drugs are used to increase sleep time; the most common group of sleep drugs are benzodiazepines that have side effects such as tolerance, withdrawal, complicated sleep behaviors and cognitive disorders in some cases. These drugs act through GABA (gamma-aminobutyric acid) receptors (Golami, 2010). Benzodiazepines (BZs) increase NREM sleep; However, BZs decreases EEG (Electroencephalography) delta power (Bastien et al., 2003 ▶) in other ways. More studies are needed to find better and more relaxing sleep drugs.

Hard envelope of date palm pollen is called spathe of *Phoenix dactylifera* (*P. dactylifera*) and it grows in the countries around the Persian Gulf. In Iranian traditional medicine, this herbal extract was used as a sedative and calmative agent. This part of date palm tree is called “Tarooneh” in Iran, “Kofarra” in Iranian ancient medical books and “Tal'e” in Arab countries. There are considerable therapeutic features reported for this herbal medicine including reducing blood fat, increasing breast milk in breast-feeding mothers, and relieving joint pain. It is also used for rheumatic diseases, as a sexual stimulant, and to relieve diarrhea and cramping. Rubbing its duff to the teeth can remove stain. It is also beneficial for gum bleeding (Dashti et al., 2012 ▶). Moreover, Dashti et al. showed the analgesic effect of this extract (Dashti et al., 2012 ▶). Also, its repellent activity against the yellow fever mosquito was shown by Demirci et al. (Demirci et al., 2013 ▶). The other effects of this extract on histological changes in testis and LH (luteinizing hormone), FSH (follicle-stimulating hormone) and testosterone concentrations were proved by Mokhtari et al (Mokhtari et al., 2007 ▶). Other research examined its effects on diarrhea and showed the anti-diarrheal effect of the extract (Al-Taher, 2008 ▶). Hamedi et al. proposed that different fractions of spathe contain different amounts of steroids, triterpene steroids, oils and flavonoids. Fourteen compounds accounting for 93% of the oil were identified in GC–MS analysis. Oxygen containing monoterpenes were the main class of components (73%) with carvacrol (37%), linalool (24%) and thymol (10%) as the major constituents of the oil (Hamedi et al., 2013 ▶). Following the previous studies on the characteristics of spathe of *P. dactylifera* as a sedative and calmative extract, this research examines the effect of hydro-alcoholic extract of spathe of *P. dactylifera* on sleep time and EEG.

## Materials and Methods

**Extracting experiment**

Phoenix dactylifera(Scientific name of date palm) is a member of Arecaceae family. Hard covering of date palm pollens were gathered from Qeshm Island (herbarium number: K1600-IMG-4924), Iran in pollination season. Then, pollens were dried and grinded into powder at room temperature. The powder was completely dissolved in 70% alcohol to be ready for pharmacological experiment. The solvent was shaken for 2 hr to be percolated. These pure solvents were rotated in rotary system to evaporate the extra alcohol from the extract. The extract was freeze-dried for 72 hr. The prepared extract was kept out of direct sunshine to avoid chemical reactions. The extract doses were chosen according to previous analgesic research that showed same analgesic and hypnotic dose-response (Shivani et al., 2014 ▶). Finally, the effects of *P. dactylifera* (tarooneh) extract (62.5, 125 and 250 mg/kg) were examined on sleep and EEG experiments. .

**Animals**

In this experiment, male Wistar rats weighting 270 ± 20g were used. The rats were provided by Isfahan University of Medical Science, Isfahan, Iran. Rats were divided into control and test groups (n=6 for each group). Control groups included intact animal (without any injection), as negative control group (saline 10 ml, i.p.) and positive control group (midazolam (Tehran Chemie Pharmaceutical co, Iran) 0.1 mg/kg, i.p.) (Koch et al., 2008 ▶). Test groups received 62.5, 125 and 250 mg/kg of the extract, i.p. For EEG experiment, saline injected in control group. Animals were kept in lucid cages in animal house at 24 ± 2 °C and humidity with 44 - 56%. Light/dark cycles of 12 hr and a reversed cycle during which the lights were turned off in day and provided a dark place for them. Then, the lights were turned on at night (19:00 pm to 7:00 am). Reversing the cycle provides better trust in sleep experiment since rats have daytime sleep-wake cycle (Vafaee et al., 2007 ▶). Rats had free access to standard rodent pellet and water, *ad libitum*.

**Sleep experiment**

Sleep was measured by behavioral Angel method (Alaei, 2001 ▶). Rat were placed in sleep physiograph system and recording started 20 min after 2-hr calming down. Four factors including sleep times, awakening time, most activity period and main sleep time interval were measured during sleep experiment. Sleeping time is duration that animals do not move.

**EEG experiment**

In this part, the rats were anesthetized by urethane (1100 mg/kg). The anesthetized rats were fixed on stereotaxic apparatus to be ready for brain surgery. In order to record brain waves, two cranial holes were drilled. The first was 1 mm anterior to bregma and 1 mm lateral to midline and the second one was drilled contralaterally 2 mm posterior to bregma and 2 mm lateral to the midline (Wursblack et al., 2014 ▶). Then, electrodes were placed subcranially for EEG recording. EEG recorder system was data acquisition system from Science Beam company, Iran that recorded EEG waves by transferring data to data acquisition system. The system processed the data to show the power of alpha, beta, delta and theta waves. Waves were filtered in range of 0 – 30 Hz. To be precise, 0.1- 3.9 Hz waves counted as delta wave, 4- 7.9 Hz waves were theta wave, 8-13 Hz waves recorded as alpha wave and 13.1 – 30 Hz waves were recorded as beta wave. Three periods of 20-min recording were recorded for each rat; in the first 20 min recording of all groups, no injections were performed and this period was considered as baseline. After the baseline, the injections were perfomed. Then, the second 20-min recording was mentioned as after 1. Before the third 20-min recording, second injection was performed and this recording period was mentioned as after 2.

**Statistical analysis**

Results are shown as mean ± SEM and statistical difference was measured using one way ANOVA with post t-test. Values were considered significant when p<0.05. Statistical analyses were performed by statistical InStat version InStat 3 software.

## Results

**Sleep experiment**

**Figure 1:**
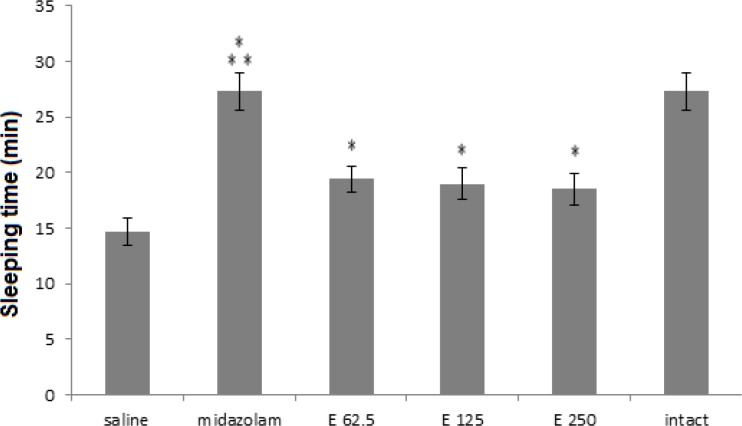
Sleeping time (minute) in 3 groups of extract treated (62.5, 125 and 250 mg/kg) and 3 control groups (saline, midazolam and without injection or intact). Midazolam and three extract groups increased sleeping time compared to saline control group. (*** p< 0.001 and * p<0.05). Each bar represents mean ± SEM

**Figure 2:**
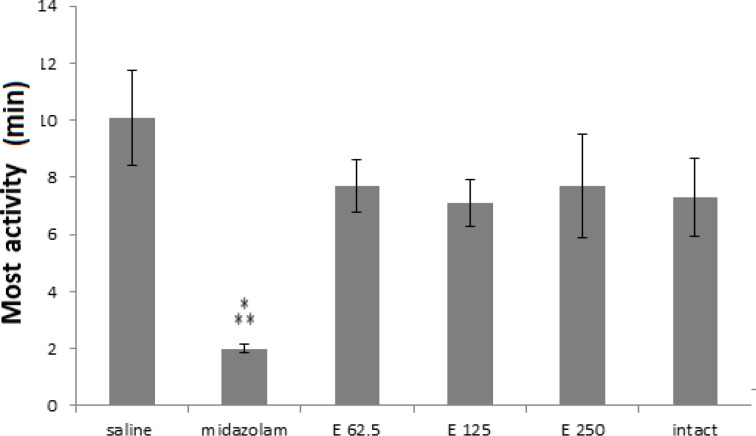
Most activities (minute) in 3 groups of extract treated (62.5, 125 and 250 mg/kg) and 3 control groups (saline, midazolam and without injection or intact). Midzolam decreased most activities in comparison to saline control group.(*** p< 0.001). Each bar represents mean ± SEM

As shown in Figure 1, in sleeping time experiment, all extract receiving groups and midazolam group were significantly different from saline group (p< 0.001) and midazolam-treated group was more significant (p<0.001). In the extract treated groups, p-values were 0.038, 0.026, 0.012 for 250, 125 and 62,5 mg/kg doses, respectively. Although three doses of the extract (62.5, 125 and 250 mg/kg) were able to increase the sleep time compared to saline control group, their effects were significantly less than midazolam (p<0.001).* In most activity experiment, all treated groups showed a decreasing trend in compare with the saline group. Meanwhile, only in midazolam treated group most activities were significantly decreased compared to the saline group (p<0.001), (Figure 2). 

**Figure 3:**
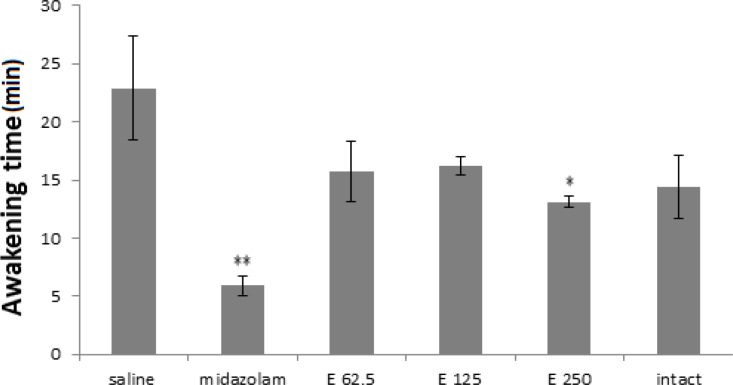
Awakening time (minute) in 3 experimental groups of extract (62.5, 125 and 250 mg/kg) and 3 control groups (saline, midazolam and without injection or intact). Midazolam and extract 250 mg/kg reduced most activity in comparison to saline control group. (** p< 0.01 and * p<0.05). Each bar represents mean ± SEM

All groups had less waking time compared to the saline group (Figure 3) but midazolam and extract 250 mg/kg groups demonstrated significant decreased waking time (p<0.01 and p<0.05 in midazolam and 250 mg/kg extract dose, respectively).

**Figure 4:**
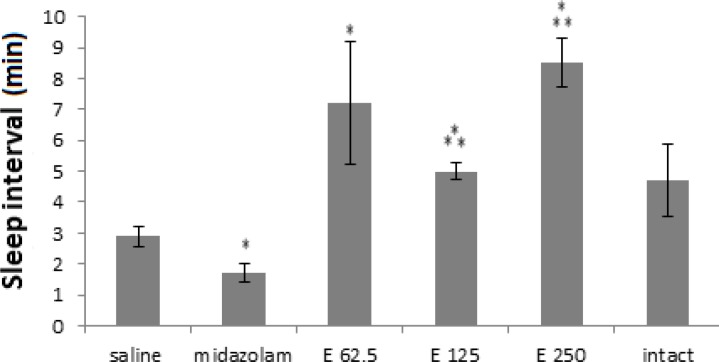
Sleep interval (minute) in 3 experimental groups of extract (62.5, 125 and 250 mg/kg) and 3 control groups (saline, midazolam and without injection). Midazolam decreased sleep distance while three extract doses increased sleeping time. (*** p< 0.001and * p<0.05). Each bar represents mean ± SEM

The main sleep time interval results were shown in Figure 4. Only midazolam was able to decline the main sleep time interval compared to the saline group (p<0.05). Meanwhile, all extract doses increased this parameter in comparison to saline group (p<0.05).

**EEG experiment**

**Figure 5:**
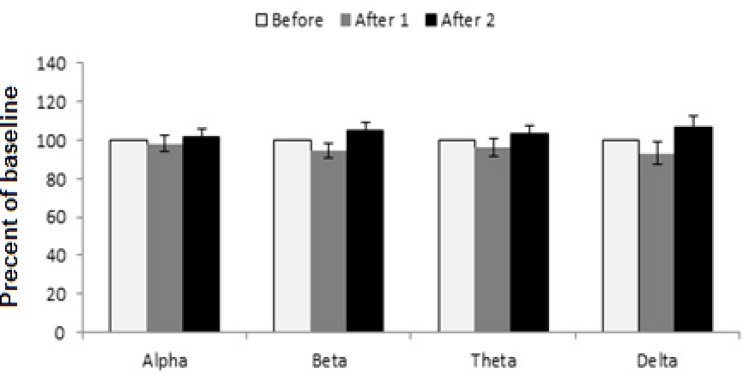
Saline injection for surveying EEG brain waves. Each bar represents mean ± SEM (white column: before wave, gray column: after 1 wave, black column: after 2 wave

**Figure 6:**
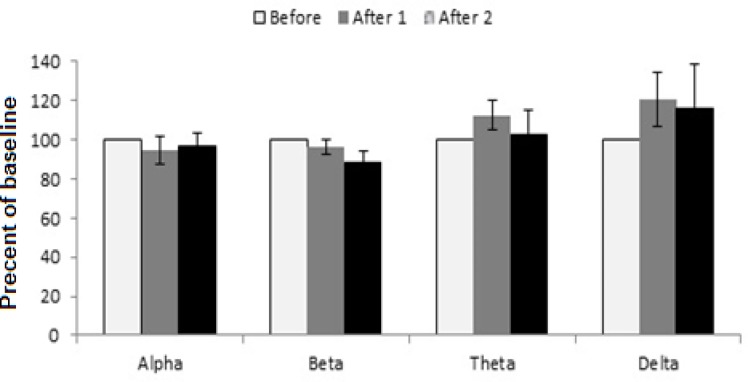
The effect of tarooneh extract 62.5 mg/kg on EEG brain waves. Each bar represents mean ± SEM (white column: before wave, gray column: after 1 wave, black column: after 2 wave

EEG results were used to achieve more details about the effect of extracts on sleep features. In Figure 5 of the saline control group, waves did not change in comparison with the before wave. However, in the extract-treated groups, these indicators were different with saline control group. At the dose of 62.5 mg/kg of the extract, alpha wave was similar to the control group while there was a small decrease in after2-beta wave, theta and delta waves increased but the changes were not significant (Figure 6). 

**Figure 7:**
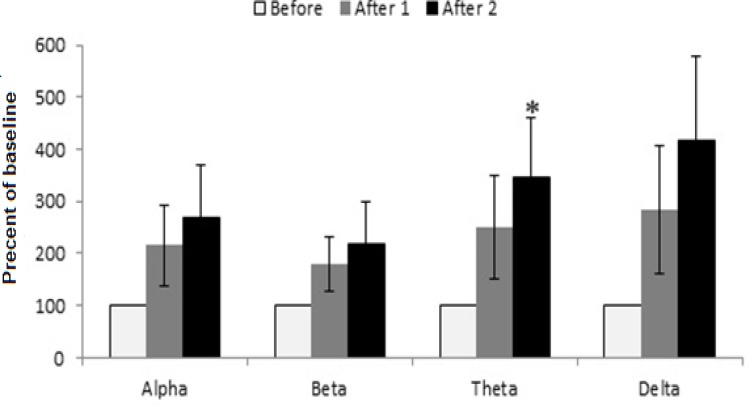
The effect of tarooneh extract 125 mg/kg on EEG brain waves. After2 theta wave was increased in comparison to its before wave, (* p<0.05). Each bar represents mean ± SEM (white column: before wave, gray column: after 1 wave, black column: after 2 wave

**Figure 8:**
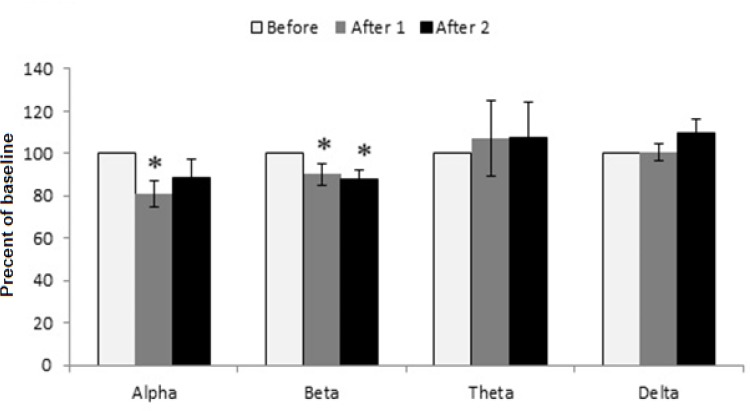
The effect of tarooneh extract 250 mg/kg on EEG brain waves. After 1 alpha wave and after1 and after 2 beta waves were decreased in comparison to their before wave, (* p<0.05). Each bar represents mean ± SEM (white column: before wave, gray column: after 1 wave, black column: after 2 wave

Before waves of dose 125 mg/kg of extract reveals changes which were significantly greater than saline and extract 62.5 mg/kg groups (Figure 7). After 1 and after 2 waves changed in comparison to the before wave. There is a significant increase in after 2 theta wave compared to before wave of dose 125 mg/kg (p<0.05) also showed increasing effect on low frequency wave of after1 theta but it was not statistically significant (p= 0.052). The extract at the dose of 250 mg/kg showed decreasing effect on after1-alpha wave in comparison to its alpha before wave, after1-beta wave was reduced as compared to before-beta wave (p= 0.050) and reduction in after 2-beta wave was similar to after 1 beta wave (p=0.018) (Figure 8).

## Discussion

The behavioral Angel method was used to examine four sleep factors. The main factor was sleeping time and the other three factors helped to clarify the effects of the extract on sleep. In sleep time experiment, all three extract doses were able to increase sleeping time although these doses showed fewer effects than midazolam. Furthermore, the intact control group which were in less stress condition, showed upward tendency for sleep time.

Most activity parameter showed reduction in all groups although the effects of midazolam were more marked which resulted in calmative effects in experimented animals. The lack of stress in without-injection (control) group caused most activity reduction.

The effects of 250 mg/kg dose of Tarooneh on sleep induction in awakening time were the same as midazolam's. Declined awakening time in intact control group was due to less stress condition. The main sleep time interval was recorded according to drowsiness and sleep before or after the main sleep. Considering other factors, there were similar changes in midazolam group, but regarding the main sleep interval factor, the extract did not show similar changes like the midazolam group. The reason of this difference is not clear but we hypothesize that this result may show that the extract can help having sufficient sleep so that after a good sleep there would not be drowsiness. Also, we can see, without-injection (control) group experienced a better sleep, there was upward tendency for sleep interval the same as the extract groups proving the first statement. Another probable hypothesis is that these doses may not be sufficient for long sleep induction. Both hypotheses need more research for confirmation. Another factor that can be studied here is the stress factor in control group. The related stress results were not effective in any of studied factors, all parameters demonstrated effects similar to midazolam except the main sleeping interval that can clarify the effects of lacking stress in sleep.

Although both saline and extract doses of 62.5 mg/kg were not effective, delta and theta waves had more ranges in group 62.5 mg/kg. The evaluation of extract at the dose of 125 mg/kg after 2 theta wave showed an increase in the high frequency power of the waves. Although only this change was significant in the theta wave, related figure of 125 mg/kg showed increasing effects on all waves compared to its baseline waves. The extract at the doses of 62.5 and 250mg/kg increased low frequency waves and decreased high frequency waves. The dose of 250 mg/kg showed reducing effects on two waves of alpha and beta which prove its sedative effect. 

Dominating one of these waves in a part of sleep exhibit the sleep stage so, these factors are good indicators. Sleep is a very important cycle in life and sleep problem can cause many difficulties (Budhiraja et al., 2011 ▶). These problems can develop into new psychiatric disorders like depression, anxiety, and substance abuse (Neuendorf et al., 2015 ▶). It is proper to find out a suitable solution for this common problem (Michal et al., 2014 ▶). Neurologic functions control sleep regulation (Cipriani et al., 2015 ▶) and it can be checked by EEG monitoring system. This method is one of the best method for monitoring of sleep (Luan et al., 2014 ▶) for showing different power waves. Scientist observed delta activity in sleep state and theta activity in less deep sleep state. Moreover, alpha activity shows calmness and beta activity is a good indicator of awareness (Lee et al., 2014 ▶).

In this research, three doses of tarooneh extract were efficient for increasing the sleeping time. However, regarding the other factors, only the dose of 250 mg/kg of the extract was significant in reducing the awakening time. In surveyed EEG experiment, the dose of 125 mg/kg of the extract showed more relaxing sleep tendency and the dose of 250 mg/kg demonstrated reduction in high frequency waves.
